# Image-Based Personalization of Cardiac Anatomy for Coupled Electromechanical Modeling

**DOI:** 10.1007/s10439-015-1474-5

**Published:** 2015-09-30

**Authors:** A. Crozier, C. M. Augustin, A. Neic, A. J. Prassl, M. Holler, T. E. Fastl, A. Hennemuth, K. Bredies, T. Kuehne, M. J. Bishop, S. A. Niederer, G. Plank

**Affiliations:** Institute of Biophysics, Medical University of Graz, Harrachgasse 21/IV, 8010 Graz, Austria; Institute for Mathematics and Scientific Computing, University of Graz, Graz, Austria; Department of Biomedical Engineering, King’s College London, London, United Kingdom; Modeling and Simulation Group, Fraunhofer MEVIS, Bremen, Germany; Non-Invasive Cardiac Imaging in Congenital Heart Disease Unit, Charité-Universitätsmedizin, Berlin, Germany; German Heart Institute, Berlin, Germany

**Keywords:** Mesh, Myocardial fiber architecture, Finite element, High performance computing, Strong scaling

## Abstract

Computational models of cardiac electromechanics (EM) are increasingly being applied to clinical problems, with patient-specific models being generated from high fidelity imaging and used to simulate patient physiology, pathophysiology and response to treatment. Current structured meshes are limited in their ability to fully represent the detailed anatomical data available from clinical images and capture complex and varied anatomy with limited geometric accuracy. In this paper, we review the state of the art in image-based personalization of cardiac anatomy for biophysically detailed, strongly coupled EM modeling, and present our own tools for the automatic building of anatomically and structurally accurate patient-specific models. Our method relies on using high resolution unstructured meshes for discretizing both physics, electrophysiology and mechanics, in combination with efficient, strongly scalable solvers necessary to deal with the computational load imposed by the large number of degrees of freedom of these meshes. These tools permit automated anatomical model generation and strongly coupled EM simulations at an unprecedented level of anatomical and biophysical detail.

## Introduction

The heart is an electrically controlled mechanical pump, which transforms chemical energy into kinetic energy. Each beat starts with the spontaneous depolarization of cells in the sinoatrial node on a timescale of milliseconds, and ends with blood flowing out of the heart to the rest of the body approximately once every second. This transduction across multiple physical systems [electrophysiology (EP), cardiac muscle mechanics, and fluid flow], multiple spatial scales (from subcellular processes to the whole cardiovascular system), and temporal scales (from fast switching of gates in the microsecond range to slower processes such as the formation and sustenance of arrhythmias on the order of seconds to minutes) makes the heart an inherently challenging organ to study through reductionist approaches. The use of biophysical models to efficiently encapsulate wider physiology and provide a simulated context for the interpretation of measured data, generate new hypotheses and predict outcomes is increasingly realized as a necessary rather than a novel element of advanced cardiac physiology and pathology studies. These models facilitate the mechanistic analysis of cause-effect relationships at high spatio-temporal resolutions in the intact organ, something not achievable with any other experimental modality.

Computational models of cardiac electromechanics (EM) are increasingly being considered in clinical applications as an additional modality to optimize therapies^42^ or understand therapy mechanisms.^43^ While detailed EP models have been used to study primarily electrophysiological diseases such as arrhythmia,^38^ other diseases such as dilated cardiomyopathy require a complete representation of EM.^43^ This growth of models beyond basic physiology into the clinic poses many opportunities for advancing patient care, but also poses challenges in the personalization of models to the complex and diverse cardiac anatomy and function in the patient population. Although every patient’s heart must achieve some common basic function, refilling and pumping blood with each beat, the variation within the population is non-negligible. This can stem from anatomical differences, for example where the right ventricular apex attaches to the left ventricle, the number of pulmonary veins, or the orientation and location of the heart within the thorax. Additional morphological and functional variations become apparent as patients age and pathologies develop with distinct scar, fibrosis, hypertrophic or dilated remodeling and cellular physiology abnormalities. Accounting for such variations requires a move away from modeling the representative heart, as has been applied in numerous animal species,^22^,^34^,^62^,^67^ towards modeling individual hearts.

In theory, this approach should be as easy as applying model creation techniques that have been developed for animal models to human cases. However, the majority of animal models were developed from work intensive and destructive *ex vivo* analysis, which is not applicable in clinical scenarios where model construction relies upon *in vivo* imaging. A number of publicly available models of the canine,^45^ rabbit^7^,^67^ and porcine^61^ cardiac anatomy have provided the anatomical basis for cardiac modeling for almost twenty years, progressing from early idealized geometric representations^45^ to more anatomically accurate models with a high level of detail.^7^

While cardiac function is often approximated as a unidirectional electro-mechano-fluidic causality chain, the coupling between the physics is bidirectional. Electrical activation and repolarization steer mechanical contraction and relaxation through excitation–contraction coupling (ECC).^6^ Any disturbances in the controlling EP acutely impair pump performance and, if they persist, trigger maladaptive remodeling processes. Conversely, alterations in mechanical environment influence EP through mechano-electric feedback (MEF),^26^ which performs important acute and regulatory roles in the adaptation of the heart’s pumping performance to metabolic demand.

While there is a clear recognition that bidirectional EM coupling is crucial to the function of the heart, this has not been reflected in the development of cardiac EM models. The vast majority of EP modeling studies ignore the effects of mechanical deformation, and most mechanical modeling studies do not explicitly represent EP as the physics controlling deformation. Most EM modeling studies have made the assumption of *weak* coupling, where EP feeds into mechanics but MEF mechanisms are not taken into account. While such models have proven suitable for addressing a variety of questions,^22^,^43^ bidirectionally or *strongly* coupled EM models are clearly preferable as there is clear evidence that EP is modulated by tissue distension.^21^,^27^

Among reasons why the majority of modeling studies preferred weakly coupled EM models, technical considerations rank highly. One major motivation for solving weakly coupled EM models has been the desire to use numerical approaches that are tailored to a specific physics, as the numerical requirements of EP and mechanics are strikingly different. EP models feature fast transients in time which translate into steep wave fronts in space. State of the art EP organ scale models are therefore discretized at high spatio-temporal resolutions to accurately capture these dynamics^9^,^44^ while also resolving fine scale structural detail^50^,^69^ and functional heterogeneities.^20^ In contrast, due to the smoother spatio-temporal characteristics of deformation^14^ numerical constraints upon discretization are less severe. Much coarser discretizations are used and fine scale anatomical features or functional heterogeneities are omitted. Furthermore, in a weak coupling scenario, EP and mechanical models can be developed independently, reducing the complexity of implementation and numerical scheme construction.

This split into two sequentially executed solution steps is reflected in a notable divergence in the employed numerical methods between EP and mechanics modeling communities. This is apparent when considering the degrees of freedom (DOF) required to discretize a human heart: in EP models DOF are on the order of tens of millions,^41^,^52^,^55^ whereas in mechanical models the DOF required are much lower, on the order of thousands^22^ to tens of thousands.^13^ To achieve sufficiently short simulation cycles in EP modeling studies, two approaches are currently being investigated: either spatio-temporally adaptive methods are employed, realized by spatial *h*-adaptivity^10^ or polynomial *p*-adaptivity,^1^ or, strongly scalable solvers are used, which reduce execution times by engaging a larger number of computational units, be it traditional CPUs^41^,^55^ or acceleration devices such as GPUs.^39^ Most mechanical modeling studies have relied upon direct solvers, which tend to be less suitable for high resolution problems.^17^ Exceptions exist where strongly scalable iterative solvers were employed, but these have only been used for vascular models.^3^,^24^

In coupled EM models a balance has to be struck between the competing demands of EP and mechanics modeling. One approach is to use overlapping meshes of different resolutions: a fine mesh for discretizing EP and a coarser mesh for mechanics.^14^,^43^ While this is readily achieved with anatomically simplified EM models,^40^ with geometrically detailed, image-based models the implementation of this approach may be more demanding under two conditions. Firstly, in cases where a perfect overlap cannot be achieved, extrapolation or projection of data between meshes will be required. Secondly, the need for the higher resolution mesh to conform to the nodes of the coarse mesh may place undue constraints on meshing and degrade mesh quality. Further, with strongly coupled EM models the computational savings may be limited, as the spatio-temporal dynamics of coupling variables impose additional spatial discretization constraints, necessitating finer spatial resolutions as it would be necessary for a weakly coupled EM problem.^47^

Alternatively, the same mesh can be used for both EP and mechanics.^15^ However, with biophysically detailed EP models, spatial resolutions <250 *μ*m are necessary to achieve acceptable accuracy.^9^,^44^ Discretizing human hearts at such resolutions gives rise to upwards of 10^8^ mechanical DOF. To deal with such vast computational loads, the use of strongly scalable iterative solvers seems necessary.^2^ Alternatively, discretization constraints can be relaxed by resorting to low dimensional EP models with slow upstroke velocities combined with simplified active stress models.^4^,^40^ This approach is less suitable for studying more subtle coupling mechanisms, as none of the key physiological quantities of interest are explicitly represented.

Despite impressive methodological advances, translating the use of computational EM models into tangible clinical benefits remains a challenging task. Construction of patient-specific anatomical models and their parameterization typically requires a complex workflow: tomographic images are acquired; image data are segmented and registered; anatomical meshes are generated^54^ and fiber architecture is mapped onto them;^5^ EP models are parameterized to approximate a patient’s electrical activation pattern and functional EP gradients to match recorded electrograms;^53^ the unstressed reference geometry is estimated and parameters describing material properties, active stresses and models of circulatory dynamics are identified based on hemodynamic data.^28^,^43^ A high degree of automation is necessary for all processing steps to minimize errors and to keep processing times within bounds compatible with clinical workflows.

While all processing stages are of high relevance, we focus this review on developments in creating personalized, anatomically accurate, computational models of coupled EM from *in vivo* imaging data, and the mapping of fiber architecture to these models for clinical applications such as cardiac resynchronization therapy in which the representation of both EP and mechanics is important. We also elucidate the corresponding computational implications regarding discretization and solving the resulting system of equations. Finally, these techniques are contrasted with a novel automatic model generation approach for high throughput modeling studies. The method is able to capture all anatomical detail that can be delineated from images with high geometric fidelity. Combined with scalable solvers for both EP and mechanics, this method enables EM modeling studies at an unprecedented level of detail without compromise of anatomical fidelity or representation of biophysical mechanisms.

## Anatomical Model Personalization

**Figure 1:**
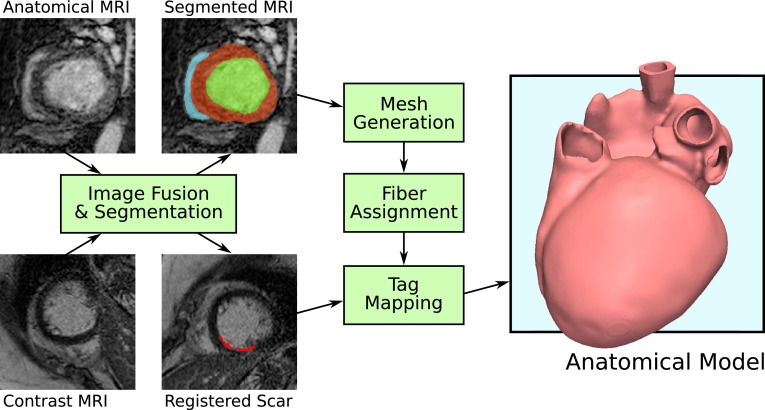
Illustration of typical workflow for generation of a personalized anatomical model of the heart from medical images. Acquired anatomical imaging, such as from MRI, is segmented, and structural or functional imaging, such as contrast-enhanced MRI for imaging scar tissue, is registered with the anatomical imaging. A FE mesh is generated from the anatomical segmentation, fiber orientations are assigned (potentially from imaging, not shown), and regional tags are mapped based on registered structural/functional data.

Medical imaging plays a pivotal role in the anatomical personalization process,^32^ as it provides both anatomical information describing the shape of a patient’s heart and structural information on fiber architecture,^64^ such as the location of scar, fibrosis, fat deposits and vascularization. The conversion of such tomographic imaging data into a discrete finite element (FE) model relies upon model generation pipelines, comprising the processing stages illustrated in Fig. 1. In practice, the accuracy of the model anatomy is limited by the resolution and quality of the source medical images, uncertainties in their segmentation, and the resolution and type of FE mesh used. In general, computed tomography (CT) offers superior resolution and contrast than Magnetic Resonance Imaging (MRI) (with resolutions on the order of 350 and 1000 * μ*m possible respectively), however the latter is more commonly used in clinical cardiology and therefore as a basis for *in vivo* computational modelling.

### Anatomical Segmentation

MRI or CT images can be segmented automatically or semi-automatically by a variety of methods, with contrast-guided region growing algorithms being popular.^68^ More advanced methods register an atlas of cardiac segmentations^71^ or a geometric model^48^ with the image to be segmented, with some using machine learning to ensure a regular and robust model fit.^70^ These techniques significantly reduce the time and propensity to operator bias of manual or semi-automatic methods. Tissue classification is also often performed at the image segmentation stage, assisting the later imposition of boundary conditions and heterogeneous assignment of electro-mechanical properties. Delineation of functionally different regions, such as fibrosis or scar, is possible by segmentation and registration of specialized imaging, as highlighted in Fig. 1.

### Anatomical Mesh Generation

The choice of method used to discretize the cardiac anatomy has significant consequences for the level of detail and accuracy of the anatomical model and for the computational cost of simulation, especially for cardiac mechanics.

#### High Order Structured Meshes for Mechanics

**Figure 2:**
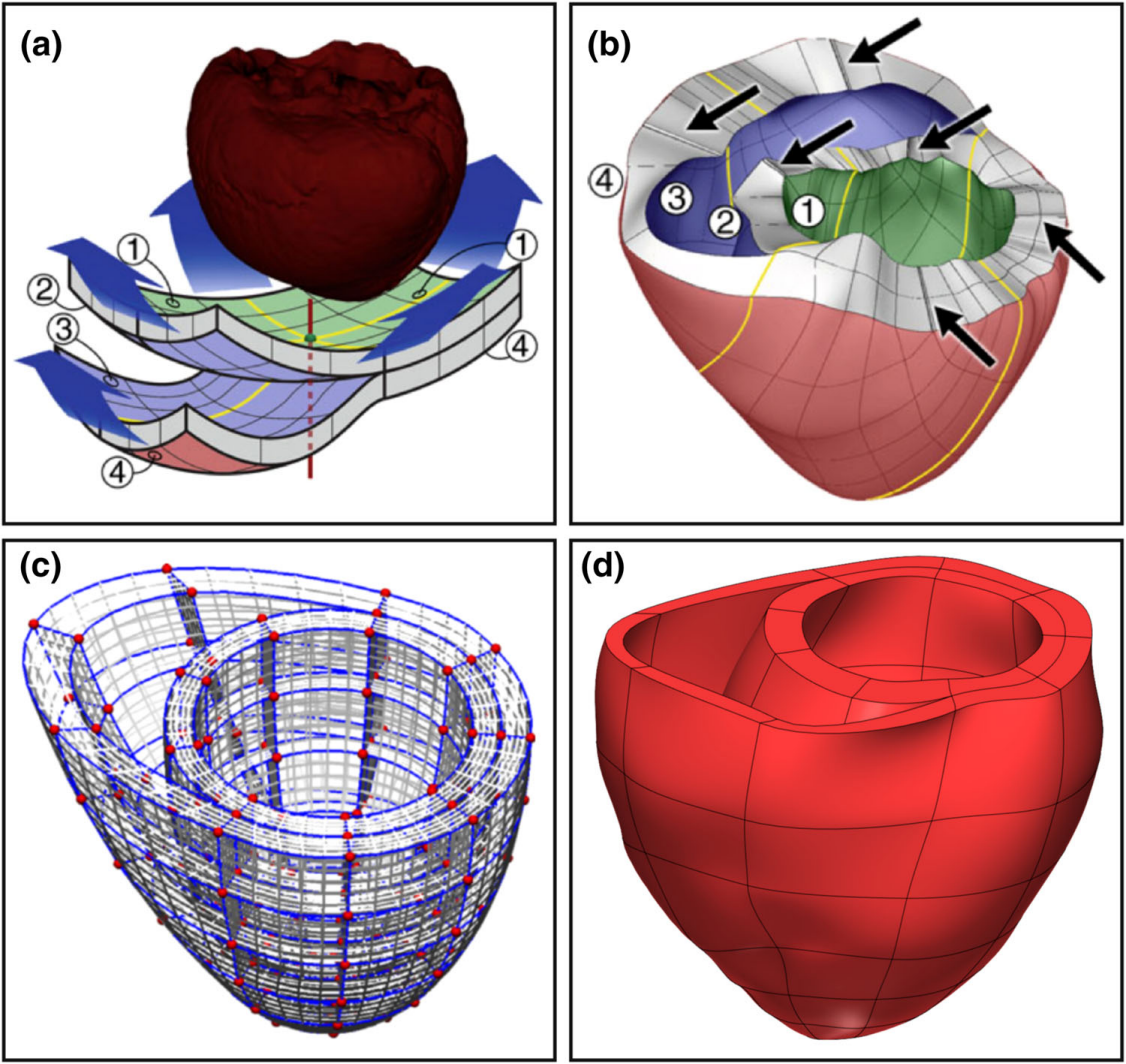
Examples of structured meshes used for anatomical modeling of the ventricles in the literature. Gurev *et al.* 14 deformed a double sheet layer with a split for the RV using a semi-automatic method (**a**) to generate a mesh personalized to the ventricular anatomy (**b**). Lamata *et al.* 30 generated a template mesh from ellipsoidal shells (**c**), which was fitted to the ventricular anatomy by an automated method utilising image registration methods (**d**).

Historically, whole organ cardiac EM models have used a weakly coupled approach, solving EP first on a fine mesh before transferring results to a coarser, high order mesh for the mechanical simulation.^14^,^43^ Structured meshes have been preferred for modeling cardiac mechanics as they facilitate geometric representations of the heart with a smaller number of elements and their regular structure often allows generating meshes of better quality. This provides computational benefits as the construction of solvers may be easier, the resulting matrices have better condition numbers, which leads to faster convergence of iterative solvers. While approaches for the image-based generation of high resolution, unstructured meshes for simulation of EP are well developed,^54^ the personalization of structured, high order meshes for the simulation of cardiac mechanics is a more difficult task. Initial personalized EM studies used a labour intensive manual manipulation approach, with additional optimization steps to improve the match with the cardiac anatomy.^14^,^43^ As illustrated in Fig. 2, later developments have enabled the semi- or fully automatic generation of such meshes, by automating the processes of mesh topology generation and template mesh alignment, combined with a robust fitting method.^30^

**Figure 3:**
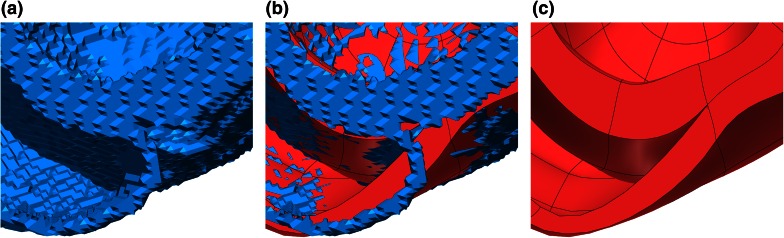
Comparison (**b**) of a tricubic Hermite anatomical model (**c**) with the source segmentation (3D isosurface,** a**) at the basoanterior join of the RV wall with the LV. The template-based cubic Hermite mesh cannot accurately capture the ventricular anatomy at joins such as this where the template does not conform to anatomical structure.

While many of the hurdles restricting the usefulness of high order structured meshes have been overcome, some fundamental limitations remain. A simplified and smoothed representation of the cardiac anatomy was advantageous when computing power was limited, however with continuing advances in hardware and numerical techniques alleviating this restriction, the smoothing cubic Hermite basis functions now restrict our ability to capture thin or fine structures such as the atria or endocardial trabeculations. Indeed, while the structured high order mesh approach has permitted the simulation of biventricular electromechanics, the right ventricular wall thickness is often overestimated for the sake of simulation stability.^33^ In addition, the use of a template based on *a priori* knowledge of the ventricular shape leads to fitting errors where the patient-specific anatomy has a different structure (Fig. 3).

#### Unstructured Meshes for Mechanics

**Figure 4:**
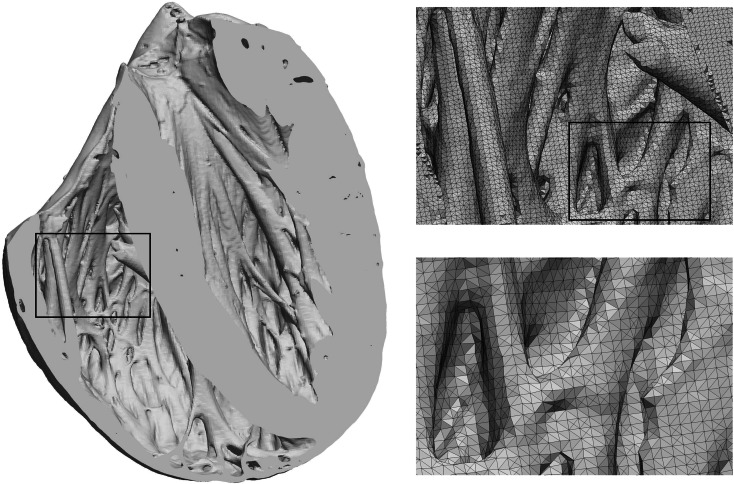
Image-based unstructured mesh generation: Shown is an anterior view of a tetrahedral FE representation of rabbit ventricles, generated from a high resolution ($\approx \!25\, \mu {\text{m}}$ isotropic resolution) *ex vivo* MRI scan.^7^,^50^ A frontal cut exposes complexity of endocardial structure such as papillary muscles and trabeculation. The mesh accounts for all geometric features which can be resolved at the chosen average mesh resolution of $\approx\! 110\, \mu {\text{m}}$.

While the use of structured meshes for modeling cardiac mechanics prevails, unstructured meshes, constructed with tetrahedral^15^,^60^ or hybrid elements,^13^ can also be used. As unstructured meshes are well established in EP modeling, mature tools are already well developed for their generation.^7^,^50^,^54^ Their key advantage is that geometrically complex objects can be automatically tessellated with smooth surface representations, including finer anatomical detail (Fig. 4). Thus the implicit smoothing and *a priori* shape assumptions of structured mesh fitting is avoided.

In principle, the same unstructured mesh can be used for the simulation of EP and mechanics. However, a mesh of sufficient spatial resolution to accurately solve EP equations with a biophysical cell model^9^,^44^ would be so large as to make the solution of mechanics computationally intractable using standard numerical software.

This problem is often circumvented by using simplified representations of cardiac EP, such as the Eikonal equation^23^,^60^ or the Fitzhugh–Nagumo model.^4^,^22^ Thus steep wave fronts associated with biophysically detailed EP models are avoided, which relaxes spatial discretization constraints and reduces computational costs. While such approaches may suffice in studies where EP serves solely as a trigger of contraction, for investigating complex EP or EM mechanisms, such as the formation and sustenance of arrhythmias^38^ or MEF effects,^35^ these models fail to capture the necessary level of detail.

Another viable approach is to use separate unstructured meshes at different resolutions for EP and mechanics. While this offers the benefit of a good accuracy to computational cost balance for both physics, it introduces a number of practical challenges in linking the simulations together, as data must be projected between two imperfectly overlapping meshes, which differ in spatial discretization and parallel partitioning.

### Fiber Architecture

Geometric models derived from imaging data describe the cardiac anatomy and the location of regional tissue variations, but do not include information regarding the distribution of fiber orientations. Architectural knowledge of the tissue’s structural anisotropy is vitally important to faithfully model electrical conduction and active force generation. However, the fiber architecture of the myocardium cannot yet be acquired clinically with a sufficiently high resolution.^64^ Modeling studies therefore determine the fiber architecture from histology or *ex vivo* diffusion tensor MRI (DT-MRI), applied to the personalized anatomical model using mathematical ‘rules’ or more complex approaches, as discussed below.

Early modelling studies used detailed histology to determine fiber orientations in the heart,^67^ with more recent application of confocal imaging permitting the delineation of fiber architecture in isolated regions of the ventricle in healthy tissue^51^ and around regions of infarct scar^56^ as well as the entire atria.^69^ DT-MRI, whilst having lower resolution than histology, as well as potential errors due to partial volume effects or changes in tissue properties following the processing of *ex vivo* samples,^16^ has the distinct advantage of providing fiber orientation information throughout the entire subject heart in a relatively efficient manner.

#### Rule-Based Methods of Assigned Fiber Architecture

The first of the so-called ‘rule-based’ methods for assigning fiber orientation to ventricular cardiac models defined a transmural variation in fiber helix angle^52^ based on histological data.^63^ Despite their simplicity, simulation studies have shown that rule-based fiber orientations produce electrical activation sequences that closely match those from models with fibers from high resolution *ex vivo* DT-MRI.^5^ However, a key limitation of these simple rule-based approaches is that they only represent fiber architecture within the bulk of the ventricular wall. Additional rules may be required to represent fiber structure within complex endocardial structures, as well as around intramural structures. Rule-based fibers may be assigned to the anatomical model by a method utilising solutions of a Laplace-Dirichlet problem to compute a local reference frame.^5^

#### Atlas-Based Methods

Atlas-based approaches are increasingly being used as a reliable means of assigning fiber architecture. In its simplest form, the fiber architecture from one heart may be directly mapped over to a new geometry.^28^,^31^,^65^ Here, a mesh warping process is used to register the geometrical mesh associated with the DT-MRI fiber data onto an idealized template mesh. Any new model requiring fiber vectors is similarly warped onto the template, and the fibers incorporated in the new model using the same variational warping technique used in the anatomical fitting process.^31^ Such single-dataset methods have been shown to successfully incorporate fiber architecture information into new image-derived geometries with no significant errors in clinically relevant electrophysiological characteristics.^31^,^65^ Atlas-based methods also have the advantage of automatically incorporating heterogeneity in fiber architecture, which may be overly complex to represent in rule-based methods.

#### Atrial Fiber Architecture

While ventricular fiber architecture has been studied extensively and numerous fiber assignment methods have been developed, less attention has been paid to atrial fiber architecture.^18^ Significant limitations of *in vivo* imaging of the thin walled atria have motivated comprehensive anatomical and morphological *ex vivo* studies.^18^,^66^,^69^

The majority of atrial modeling studies have incorporated fiber architecture using rule-based approaches, where the defined rules qualitatively approximated reports in the literature. However, due to the complex nature of atrial fiber architecture, deriving a set of rules sufficiently generic to be applicable to the entire atria remains a challenge; rather, rules are generally assigned manually to specific atrial regions.^11^,^58^ Various rule-based approaches have been proposed, all of which require varying degrees of manual intervention.^29^,^57^ More recently, atlas-based methods using a number of distinct landmarks have been proposed.^37^,^57^ Modeling studies which accounted for the complex atrial fiber architecture demonstrated its influence on both atrial EP and mechanics.^29^,^57^,^69^

## High Resolution Whole Organ EM Model Generation Pipeline

The modeling of strongly coupled EM poses a particular challenge for the discretization of the solution domain. Historically, studies have used coarse, high order meshes for simulating mechanics to keep computational costs low, though other practical considerations limit the coupling of such a model to spatially converged EP.

We have therefore developed a simulation software capable of simulating whole organ mechanics at a high spatio-temporal resolution, so that both EP and mechanics can be solved, and spatially converged, on the same grid.^2^ Not only does this alleviate the practical problems of projecting information between computational meshes of complex topolgies and different resolutions, but it also enables the automatic generation of anatomical models for EM from medical images using existing mesh generation tools^54^ with a high geometric fidelity. Building on these tools, we have developed a robust pipeline for the generation of personalized models of cardiac EM from clinical imaging. Except for the initial segmentation stage which requires interactive processing, the entire model building workflow is fully automatable.

### Model Generation

#### Image Segmentation

In our model generation pipeline, the cardiac anatomy is first segmented from source medical imaging by one of the semi- or fully automatic methods discussed above. The segmentation is tagged by anatomical region, assisting the later imposition of boundary conditions and regional differences in electrical and mechanical material properties.

**Figure 5:**
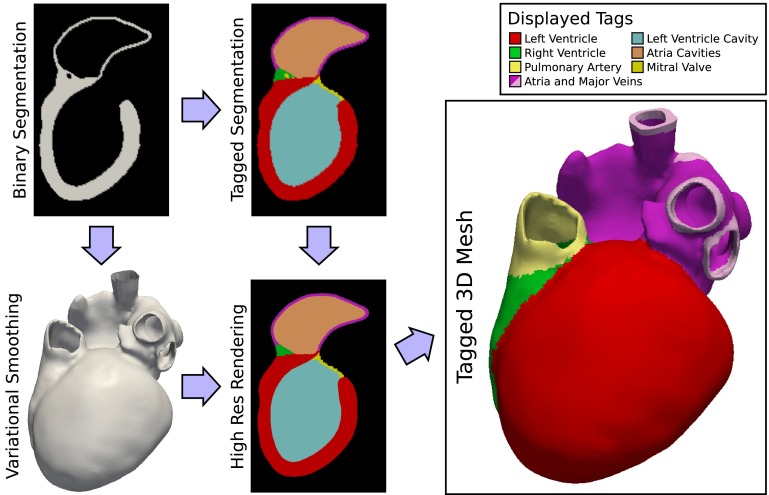
Workflow for the generation of tagged, high resolution models of the cardiac anatomy from a medical image segmentation. The segmentation is tagged by anatomical region and separately smoothed by a variational method. The smoothed surface is re-rasterized at a high resolution and regional tags are mapped to the new image stack. This image stack is finally fed into an image-based mesh generator to construct a high resolution, tagged, 3D anatomical model which closely matches the source segmentation.

The anatomical model shown in Fig. 5 illustrates our anatomical model processing pipeline. The dataset shown was derived from a whole heart, end diastolic, 3D, steady state free precession (SSFP) MRI with an isotropic resolution of 1.3 mm. Segmentation was performed by a model-based method.^48^

#### Segmentation Smoothing and Upsampling

Prior to the generation of a FE mesh, a smoothing and upsampling step is performed on the segmented image stack . The upsampling step, which increases spatial resolution from clinical resolution ($\approx \!1$ mm) to modeling resolution ($\approx \!100\,\mu {\text{m}}$) is performed to avoid ‘staircase’ effects that occur when meshes are generated at a much higher resolution than the source segmentation, as the mesh traces the boundaries of the relatively large voxels of the segmentation. A combined smoothing and upsampling step attenuates this effect, resulting in a significantly improved representation of myocardial surfaces in the model.

Starting from a lower resolution, anatomically tagged image segmentation, we generate a three dimensional surface mesh delineating the boundaries between anatomical tags. A binary segmentation of each tag is created, and its bounding surface is triangulated using the marching cubes method.^36^ The resulting surfaces are combined, with redundant interfaces removed.

A variational method^25^ is then employed to correct for low resolution artifacts of the surface. Smoothing is achieved by minimizing a high order penalty,^8^ in this case the quadratic norm of the Laplacian of the surface nodes, subject to neighborhood box constraints imposed on surface nodes. The maximum displacement of the surface from its initial state is restricted to $\pm 0.5$ of the voxel size, ensuring that the result is within the margin of error of the segmentation. The resulting smoothed surface representation is then rendered, generating a new tagged image segmentation of arbitrary resolution.

#### Mesh and Fiber Generation

**Figure 6:**
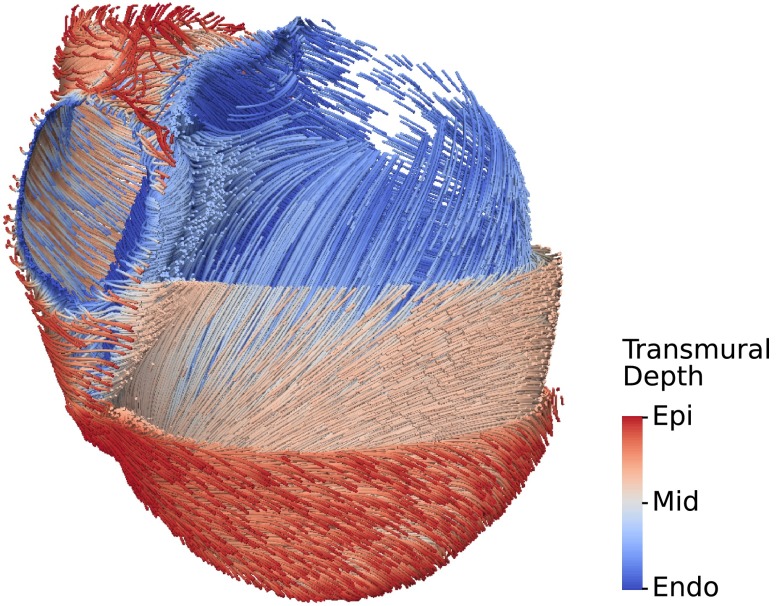
Visualization of fiber structure generated by a rule-based method.^5^ Shown are the principal fiber direction in the ventricles, with layers of the LV cut away to reveal the transmural variation.

A high resolution mesh of a four chamber heart is created using the Tarantula mesh generation software (CAE Software Solutions, Eggenburg, Austria), which builds unstructured, boundary fitted, locally refined tetrahedral meshes^54^ and maps classification tags from the input segmentation onto the generated mesh. Orthotropic eigenaxes are assigned in both ventricles using the Laplace-Dirichlet rule-based method.^5^ This method requires the selection of LV endocardium, RV endocardium and biventricular epicardium, plus apex and base of the heart. These selections are automatable using the assigned classification tags by extracting surfaces of individual tag sets and performing logical set operations on these surfaces. For instance, the epicardial surface, $\Gamma _\text {epi}$, is found as the combination $\Gamma _\text {epi} = \Gamma _\text {LVepi} \cap \Gamma _\text {RVepi}$, where $\Gamma _\text {LVepi}$ and $\Gamma _\text {RVepi}$ are RV and LV epicardium, respectively, which in turn are found as the intersections $\Gamma _\text {LVepi} = \Gamma _\text {LV} \cup \Gamma _\text {B}$ and $\Gamma _\text {RVepi} = \Gamma _\text {RV} \cup \Gamma _\text {B}$, where $\Gamma _\text {LV}$, $\Gamma _\text {RV}$ and $\Gamma _\text {B}$ are the surfaces of the tag sets RV, LV and background. Figure 6 shows the generated fiber orientations.

### Simulation Results

**Figure 7:**
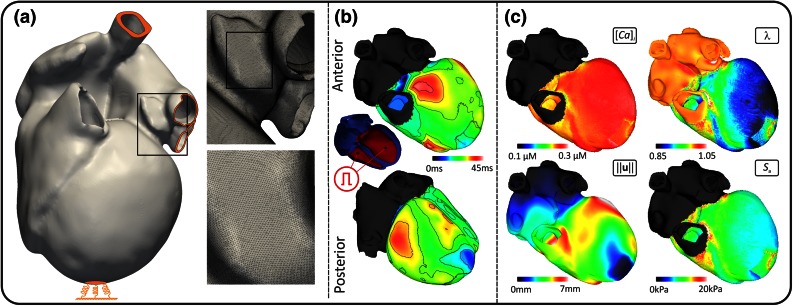
(**a**) Four chamber heart model discretized at a spatial resolution of $220\,\mu {\text{m}}$. Dirichlet boundary conditions were applied at the termini of the meshed superior and inferior cavae, all pulmonary veins and at the bottom of a soft material block attached to the apex (*orange*). Insets illustrate geometric detail and smoothness of the discretized model. (**b**) Electrical activation was initiated by stimulating the ventricular endocardia. Local activation times are shown from anterior and posterior views. (**c**) An entire heart beat was simulated over 500 ms. The spatial distribution of intracellular calcium $[\text{Ca}^{2+}]_{\text{i}}$, fiber stretch ratio $\lambda$ and displacement norm $||{\mathbf {u}}||$ at $t=130\,{\text{ms}}$, and active stress $S_{\text {a}}$ at $t=190\,{\text{ms}}$ are shown. Due to strong coupling, where calcium binding of troponin C is a function of stretch, heterogeneity in $\lambda$ is reflected in $[\text{Ca}^{2+}]_{\text{i}}$.

Feasibility of our approach is demonstrated by simulating a heart beat of a human Langendorff setup using the high resolution four chamber anatomical model illustrated in Figs. 5 and 6. The model was discretized at an average resolution of $220\,\mu {\text{m}}$, yielding a mesh of 184.6 million tetrahedral elements and 95.9 million displacement DOF (Fig. 7a). The cost of solving the large systems of equations was addressed by developing a highly parallel, strongly scalable a domain decomposition algebraic multigrid preconditioner for an iterative Krylov solver,^39^,^49^ adapted for nonlinear biomechanics.^2^ The solver converged on avarge in $\approx$6 Newton iterations with an average number of $\approx$250 iterations per linear solver step. The bidomain equations were solved as described previously.^39^ The same mesh was used for discretizing both EP and mechanics equation. Cellular dynamics was represented by the Grandi–Pasqualini–Bers human ventricular myocyte model,^12^ strongly coupled to the Land–Niederer active stress model,^34^ with the orthotropic Holzapfel–Ogden constitutive model.^19^ Spatial distribution of intracellular calcium $[\text{Ca}^{2+}]_{\text{i}}$, fiber stretch $\lambda$, displacement norm $||{\mathbf {u}}||$ and active stress $S_\text {a}$ are shown in Fig. 7c. The simulation of a single heartbeat took 235.3 minutes using 8 192 compute cores on the SuperMUC high performance computing (HPC) resource.

## Future Directions and Challenges

Developments in the field of personalized EM modeling are primarily focused on the drive towards clinical utility and application of models in an advanced diagnostic workflow. While personalized models have been used in a single case to investigate problems of clinical interest,^42^,^43^ the complex and labor-intensive process of generating models from clincal data restricts their application as a regular diagnostic or treatment planning tool in the clinic, where relatively fast turnaround times are required. While advances in the tools and processing pipelines for model building, as discussed in this paper, have made significant progress towards this goal, further streamlining is required.

Computer models are inherently approximations and are not able to reliably capture every aspect of cardiac function. A key component in modeling the heart are the cellular models that couple EP, calcium handling and contraction. Current models are built by combining multiple existing models, but the increased need for well described coupled cell models for organ scale simulations will hopefully lead to dedicated coupled models in the future. Such models allow us to simulate cardiac cells under physiological loading conditions to improve determining the parameters for cellular model under in-vivo physiological conditions. Including the interaction between EP and mechanics will move us closer to simulating the complex regulation and physiology of the heart.

Beyond building anatomical models, the parametrization of EM models as well as their validation and verification is an open challenge^46^ that needs to be addressed to gauge the reliability of model predictions. However, most model parameters cannot be measured *in vivo* with sufficient accuracy, or they cannot be measured at all and have to be identified, something that is not feasible today for EM models in a unique manner. The development of robust data assimilation strategies is therefore of utmost importance.^59^

Computations in cardiac EM modeling software are increasingly being performed on accelerators such as GPUs,^39^ which opens up exciting new directions for the possible applications of EM modeling software. High resolution models of whole organ function, as discussed in this paper, must be computed on HPC resources, which due to their expense and size are usually shared and off-site. Particularly when combined with simpler models with less demanding spatial resolution requirements, such as the Eikonal equation, GPU computation permits rapid computation of a personalized EM model on hardware of a size and price compatible with on-site deployment in a clinical setting.

## Conclusions

Over the past decade, a number of effective workflows have been developed for creation of patient-specific, anatomically accurate EM models. Template-based automatic structured mesh generation techniques can provide personalized biventricular anatomies with few DOF, but a significant degree of geometric simplification is inevitable and exact anatomical correspondence cannot be guaranteed.^33^ Conversely, fully automatic, image-based unstructured meshing techniques have reached a level of maturity that enables the generation of large cohorts of models in high throughput modeling studies without compromising geometric fidelity.^54^

The disadvantage of this latter approach is the large number of DOF incurred by a discretization which is sufficiently fine for the computation of nearly converged solutions^9^,^44^ when considering biophysically detailed strongly coupled EM models. While the use of such a high resolution is accepted as necessary for modeling EP, this is not yet the case for mechanics. However, computational limitations are steadily being alleviated as more and more powerful hardware becomes available in the era of Exascale computing and better scalable numerical methods are developed.^2^,^24^

These technologies are poised to enable a new cardiac EM modeling paradigm in which cardiac anatomy is represented with high geometric fidelity, and where the level of biophysical detail is chosen according to the questions being addressed, without the tight technical limitations of weakly coupled EP and mechanics modeling tools.
